# Alcohol: Its Place in Medical Practice

**Published:** 1910-05

**Authors:** 


### Alcohol: Its Place in Medical Practice.

Dr. W. Waugh in a paper read before the American Society for the Study of Alcoholism and Other Narcotics at Atlantic City says, "In discussing this question, I have left out absolutely all consideration of the moral effects and perils accruing from the use of alcohol, and have endeavored to look at the question simply from the standpoint of a physician who is anxious to know what is the best treatment to be given to his patient in each condition of disease the doctor is called upon to treat.

SUMMARY.

1. As a substitute, strychnine excels alcohol as a vital stimulant and an energizer of all the vital functions.

2. In shock, syncope and other forms of cerebral anemia, the combination of glonoin and atropine is quicker, safer and genuinely effective.

3. To quiet nervous apprehension and enable the patient to look with equanimity upon his condition, without embarrassing the surgeon by his dread of anesthetics, or of a proposed operation, an injection of morphine and hyoscine is much more effective than any quantity of alcohol that could be given, without adding to the danger of the condition, as alcohol would certainly do.

4. As a stimulant digestian alcohol is not equal to quassin or other simple bitters, with such artificial digestants as the case may require.

5. To prevent a cold or chill when wet, alcohol is not equal to a hot drink containing camphor or spice, or to a hot mustard foot-bath.

6. As a remedy for pain, nobody would think of using alcohol, excepting in the absence of morphine, hyoscine, ether, chloroform, atropine, camphor, cannabis, or any of the other direct analgesants.

7. In all forms of diarrhea and dysentery it is now understood that the best treatment is to remove the cause or irritation, and to soothe the irritated pneumogastric by the use of atropine, stopping microbic action in the alimentary canal by the use of intestinal antiseptics, and in case of dysentery soothing the irritation by single doses of emetine. There is no place for alcohol in this group of diseases in the hands of those who know how to use the active remedies of our profession.

8. As a hypnotic, alcohol may produce sleep by paralyzing the cerebral functions of the patient, a most undesirable and irrational method; or by momentarily equalizing the cerebral vascular pressure. In the latter case the same effect may be safely and quickly induced by administering a glass of hot water, or a few granules of aconitine, or of digitalion, according as the pulse tension needs to be lowered or elevated. Here again alcohol could only be used by those ignorant of the rescurces of modern medicine, and incapable of recognizing a pathologic condition and applying to it the remedy best calculated to restore normal, physiologic equilibrium. This applies to every use that could be devised for alcohol in the treatment of disease.—*The Dietetic and Hygienic Gazette.*

